# Notes and News

**Published:** 1893-10-28

**Authors:** 


### Notes and News.

Collected and Communicated.

The following table shows the number of students who have commenced the study of medicine at the several schools.

| | A. | B. | C. | D. | E. |
|---|---|---|---|---|---|
| St. Bartholomew's Hospital | 95 | 33 | 0 | 28 | 128 |
| Charing Cross Hospital | 20 | 12 | 25 | 1 | 57 |
| St. George's Hospital | 21 | 3 | 0 | 0 | 24 |
| Guy's Hospital | 116 | 58 | 32 | 32 | 206 |
| King's College | 33 | 74 | 0 | 12 | 107 |
| London Hospital | 72 | 76 | 1 | 9 | 149 |
| St. Mary's Hospital | 79 | 77 | 0 | 8 | 156 |
| Middlesex Hospital | 30 | 22 | 11 | 6 | 63 |
| St. Thomas's Hospital | 71 | 15 | 0 | 8 | 86 |
| University College | 43 | 46 | 0 | 75 | 164 |
| Westminster Hospital | 18 | 6 | 0 | 0 | 24 |
| London School of Medicine for Women | 25 | 16 | 0 | 0 | 41 |
| Cambridge University | 132 | 0 | 0 | 0 | 132 |
| University of Durham College of Medicine | 43 | — | — | — | — |
| Bristol Medical School | 16 | 0 | 0 | 20 | 16 |
| Owens College, Manchester | 73 | 39 | 7 | 59 | 119 |
| University College, Liverpool | 29 | 0 | 4 | 11 | 33 |
| Yorkshire College, Leeds | 41 | 0 | 0 | 0 | 41 |
| Mason College, Birmingham | 36 | 38 | 7 | 13 | 81 |
| London School of Dental Surgery | 0 | 0 | 45 | 0 | 45 |

A. Number of students who have entered for the full curriculum.
B. Number of students who have joined for special courses.
C. Number of dental students.
D. Number of students who have joined classes for preliminary scientific instruction.
E. Total excluding students who have joined classes for preliminary scientific instruction.

Guy's Hospital has cause to be satisfied with the results secured to this ancient school by the enterprise of its present managers. We are delighted to find that this year it takes its proper place at the head of the medical schools. Next in order come University College, St. Mary's, the London and St. Batholomew's Hospitals amongst London schools. Whilst Cambridge University and St. Mary's assume a position of prominence worthy of the splendid energy which has long characterised the conduct of their Medical Schools. The entry as a whole is remarkable, owing to the large number of new students, despite the added expense and strain entailed by an extra year's curriculum.

The hundredth anniversary of John Hunter's death was celebrated at St. George's Hospital by a large soirée, at which about 800 guests were present. The event was made the occasion for an exhibition of a most interesting nature, and many portraits of Hunter and his connections were shown.

The Metropolitan Asylums Board have actually surmounted their difficulties to the extent of establishing another fever hospital. Their new hospital at Grove Road, Lower Tooting, promises to be open in a few days. It will accommodate 406 patients, and it is proposed to retain it at present solely for scarlet fever patients. The estimated cost is about £60,000.

Mr. Passmore Edwards, who had formed the generous intention of presenting West Ham with a cottage hospital, has now decided that to extend the present hospital would be more beneficial to the neighbourhood. He will therefore devote £2,000 towards the erection of a new wing. It is hoped that the Prince of Wales may be induced to lay the foundation-stone next month.

Investigations of the charges made against the management of the West Ham Hospital are being carried on before the Town Council. The fact that the meetings have been held *in camera* has caused much dissatisfaction. The *Morning*, the paper which has been principally concerned in the attack, makes one statement which, although it is not intended as such, is reassuring. It says that a great improvement has taken place in the hospital during the last few months, and that recent inmates are therefore not suitable witnesses against the state of affairs complained of. This is rather as if reform without the drawing of blood were unacceptable, and that present improvement could not wipe out past sins, a matter that seems very undesirable to those who would see useful work done in any institution.

An animated meeting has been held at Norwood on the matter of the Hospital Saturday Fund frauds there. The frauds were discovered by the Rev. Walter Hobbs, his action being censured by the Hospital Saturday council, who maintain he has placed grave difficulties in their way by his mode of procedure. Dissatisfaction with the returns of the monies collected in the district it appears is by no means a new departure, having in fact extended over years. Therefore, the conduct of Mr. Hobbs is to be commended, whilst the behaviour of the executive, and of Mr. Allen, secretary and responsible official, is, to say the least, not beyond question. On the occasion of the inquiry, Mr. Allen was not present to give an explanation of the grave charges made. The result of the meeting made it very clear that the receipts given by the secretary, Mr. Allen, did not tally with the amount known to be in the boxes handed in by individual collectors. Several speakers at the meeting expressed a wish that the inquiry would result in renewed prosperity to the Hospital Saturday collection. The best way of securing such a result will be to institute a prosecution in the present instance, and to abolish all street collections for the future.

